# The miR-30-5p/TIA-1 axis directs cellular senescence by regulating mitochondrial dynamics

**DOI:** 10.1038/s41419-024-06797-1

**Published:** 2024-06-10

**Authors:** Hyosun Tak, Seongho Cha, Youlim Hong, Myeongwoo Jung, Seungyeon Ryu, Sukyoung Han, Seung Min Jeong, Wook Kim, Eun Kyung Lee

**Affiliations:** 1 Department of Biochemistry, The Catholic University of Korea, Seoul, 06591 South Korea; 2 INSERM U1052, CNRS UMR-5286, Cancer Research Center of Lyon (CRCL), Lyon, 69008 France; 3 Department of Biomedicine & Health Sciences, The Catholic University of Korea, Seoul, 06591 South Korea; 4 Institute for Aging and Metabolic Diseases, College of Medicine, The Catholic University of Korea, Seoul, 06591 South Korea; 5 Department of Molecular Science & Technology, Ajou University, Suwon, 16499 South Korea

**Keywords:** miRNAs, Senescence, Mechanisms of disease

## Abstract

Senescent cells exhibit a diverse spectrum of changes in their morphology, proliferative capacity, senescence-associated secretory phenotype (SASP) production, and mitochondrial homeostasis. These cells often manifest with elongated mitochondria, a hallmark of cellular senescence. However, the precise regulatory mechanisms orchestrating this phenomenon remain predominantly unexplored. In this study, we provide compelling evidence for decreases in TIA-1, a pivotal regulator of mitochondrial dynamics, in models of both replicative senescence and ionizing radiation (IR)-induced senescence. The downregulation of TIA-1 was determined to trigger mitochondrial elongation and enhance the expression of senescence-associated β-galactosidase, a marker of cellular senescence, in human foreskin fibroblast HS27 cells and human keratinocyte HaCaT cells. Conversely, the overexpression of TIA-1 mitigated IR-induced cellular senescence. Notably, we identified the miR-30-5p family as a novel factor regulating TIA-1 expression. Augmented expression of the miR-30-5p family was responsible for driving mitochondrial elongation and promoting cellular senescence in response to IR. Taken together, our findings underscore the significance of the miR-30-5p/TIA-1 axis in governing mitochondrial dynamics and cellular senescence.

## Introduction

Cellular senescence, a state of irreversible growth arrest, is a complex process characterized by distinct morphological and functional changes in cells [1–3]. Various stressors, including DNA damage, telomeric shortening, oncogene activation, epigenetic alteration, and mitochondrial dysfunction, trigger cellular senescence [4]. Senescent cells display flattened and enlarged morphology and enhanced senescence-associated β-galactosidase (SA β-gal) activity. They exhibit signs of DNA damage that result in increased levels of p53 (encoded by *TP53*), p21 (encoded by *CDKN1A*), p16 (encoded by *CDKN2A*), and phosphorylation of histone variant H2AX (γH2AX) [5]. Senescent cells lose their ability to proliferate, but they are metabolically active and secrete a variety of molecules, collectively termed the senescence-associated secretory phenotype (SASP), thereby influencing neighboring cells and the tissue microenvironment [6, 7]. Although senescence is necessary for normal developmental processes and wound healing, it exerts deleterious effects by promoting tumor development, stem cell exhaustion, and chronic inflammation [8]. In addition, the aberrant accumulation of senescent cells in aged tissues suggests that senescence is a hallmark of aging and can drive aging and age-related diseases [5]. Therefore, elucidating the molecular mechanisms underlying cellular senescence in various circumstances is essential to broadening our knowledge of aging and the pathogenesis of age-related diseases.

Mitochondrial dysfunction has been suggested as one of the drivers of cellular senescence [9–11]. It contributes to cellular senescence by impairing energy production, increasing reactive oxygen species (ROS), accumulating mitochondrial DNA (mtDNA) damage, and disrupting mitochondrial signaling and dynamics [11–13]. Mitochondrial dynamics, essential for mitochondrial homeostasis, are governed by fission (fragmentation) and fusion (elongation) mediated by several regulatory proteins, including dynamin-related protein 1 (DRP1), mitochondrial fission 1 (FIS1), mitochondrial fission factor (MFF), mitofusin 1/2 (MFN1/2), and optic atrophy 1 (OPA1) [14]. Several reports showed that impairing mitochondrial dynamics and aberrant levels of those regulatory proteins contributed to cellular senescence [15–18]. In addition, various types of senescent cells or aged tissues exhibit abnormal mitochondrial dynamics and have hyper-elongated giant mitochondria [11, 19–21]. Although detailed mechanisms need to be defined, these findings suggest a strong correlation between mitochondrial dynamics and cellular senescence.

Based on prior investigations conducted by our group and other scholars, which underscored the role of the RNA-binding protein TIA-1 in governing mitochondrial dynamics [22, 23], we initiated an investigation to determine whether the TIA-1-mediated regulation of mitochondrial dynamics could impact cellular senescence. We observed an increase in mitochondrial elongation and a decrease in TIA-1 expression across various types of senescent cells. A series of experiments consistently demonstrated that the downregulation of TIA-1 led to both mitochondrial elongation and cellular senescence. The knockdown of TIA-1 promoted stress-induced cellular senescence following ionizing radiation (IR) exposure, whereas the induction of TIA-1 through ectopic expression mitigated this effect. Further analysis aimed at uncovering the cause of TIA-1 reduction in senescent cells identified the microRNA-30-5p (miR-30-5p) family as a pivotal factor. In particular, miR-30-5p, upregulated in several senescence or aging models, was found to be accountable for the reduction in TIA-1 expression during cellular senescence. The inhibition of miR-30-5p demonstrated a beneficial effect, alleviating TIA-1 depletion-associated mitochondrial elongation and cellular senescence. In summary, our findings suggest that a decrease in TIA-1 expression contributes to the enhancement of cellular senescence, and the miR-30-5p/TIA-1 axis emerges as an innovative modulator within the context of cellular senescence.

## Results

### Senescent cells have elongated mitochondria

To investigate the correlation between cellular senescence and mitochondria morphology, we analyzed the relative mitochondrial length in various models of cellular senescence, including replicative and stress-induced senescence. Cellular senescence was evaluated by staining and quantifying SA β-gal expression, while mitochondrial morphology was ascertained by measuring the relative length of mitochondria in images of cells stained with MitoTracker.

**Fig. 1:**
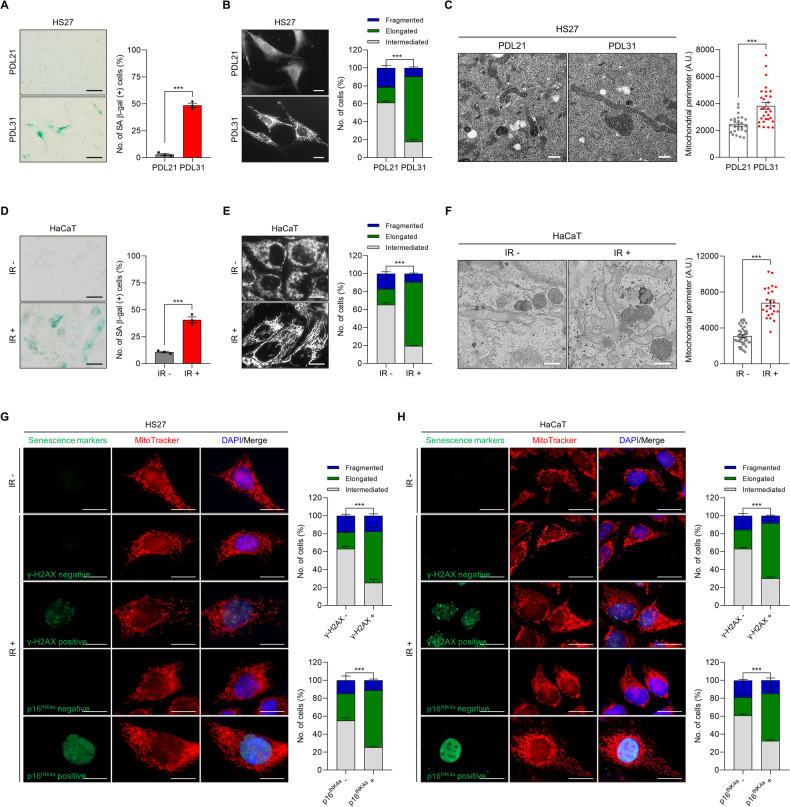
Enhanced mitochondrial elongation in senescent cells. **A** SA β-gal analysis of young (PDL21) and senescent (PDL31) HS27 human skin fibroblasts. **B**, **C** Mitochondrial morphology in young and senescent HS27 cells. **D** SA β-gal analysis of HaCaT human keratinocytes with or without IR exposure (6 Gy, 72 h). **E**, **F** Mitochondrial morphology in HaCaT cells. For SA β-gal analysis, cells were incubated with a staining solution (pH 6.0), and the number of SA β-gal-positive cells was counted. Mitochondrial morphology was assessed by staining with MitoTracker (100 nM) and transmission electron microscopy. The number of cells with fragmented, elongated, or intermediated mitochondria was analyzed using Image J software. Quantitation of the mitochondrial perimeter was calculated from more than 25 mitochondria in each experimental group using Image J software. **G**, **H** Immunofluorescence microscopy of HS27 and HaCaT cells co-stained with γH2AX, p16^INK4a^, and MitoTracker with or without IR exposure (6 Gy, 72 h). Images are representative, and data are presented as the mean ± SEM of three independent analyses. Scale bar, 20 μm (**A**, **B**, **D**, **E**, **G**, **H**) and 0.5 μm (**C**, **F**). ***p < 0.001.

Replicative senescence was induced through the continuous passaging of human foreskin fibroblast HS27 cells in culture, resulting in an increase in SA β-gal expression (Fig. 1A). Senescent HS27 cells (Population Doubling Level (PDL) 31) exhibited notably elongated mitochondria compared to their young counterparts (PDL 21) (Fig. 1B, C). Additionally, exposure to ionizing radiation (IR), a potent inducer of cellular senescence, increased SA β-gal levels in human fetal lung fibroblast WI-38 and transformed human keratinocyte HaCaT cells (Figs. S1 and 1D). Notably, IR exposure also increased the relative length of mitochondria in both cell lines (Figs. S1, 1E, F). Furthermore, exposure to IR not only led to the forming of γH2AX-positive foci within cells or induction of p16^INK4a^ but also concurrently induced mitochondrial elongation in these identical cells relative to their non-senescent counterparts (Fig. 1G, H). Collectively, these results indicate that senescent cells tend to possess more elongated mitochondria.

### Senescent cells have reduced TIA-1 expression

**Fig. 2:**
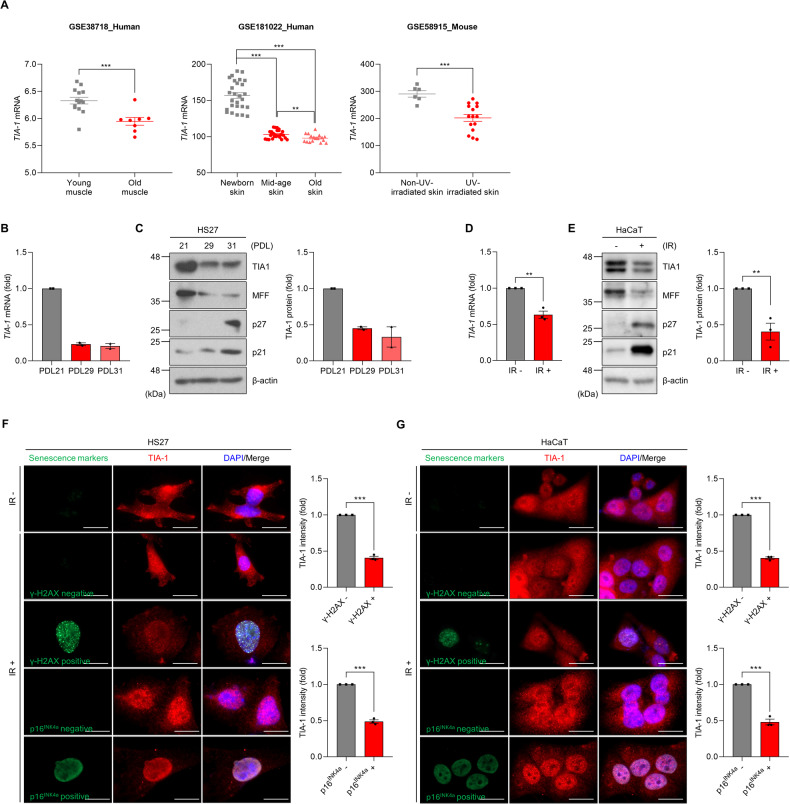
Downregulation of TIA-1 expression during cellular senescence. **A** Relative levels of *TIA-1* mRNA in several Gene Expression Omnibus (GEO) datasets (GSE38718, GSE181022, and GSE58915). **B**, **C** *TIA-1* mRNA and protein levels in HS27 cells at various PDLs. **D**, **E** *TIA-1* mRNA and protein levels in HaCaT cells with or without IR exposure. *TIA-1* mRNA was analyzed by RT-qPCR. *GAPDH* mRNA was used as a reference gene for normalization. Protein expression was determined by Western blotting (WB) and quantified by densitometric analysis using Image J software. β-actin was used as the loading control. **F**, **G** Immunofluorescence microscopy of HS27 and HaCaT cells co-stained with γH2AX, p16^INK4a^, and TIA-1 with or without IR exposure (6 Gy, 72 h). Fluorescent signals against of TIA-1 in each experimental group were analyzed using Image J software and normalized with cell numbers. Images are representative, and the data are presented as the mean ± SEM of two (**B**, **C**) or three (**D**–**G**) independent analyses. Scale bar, 20 μm. **p < 0.01, ***p < 0.001.

TIA-1 has been shown to regulate mitochondrial dynamics. TIA-1 knockdown led to mitochondrial elongation, whereas TIA-1 overexpression promoted mitochondrial fragmentation [22–24]. These findings prompted our hypothesis regarding decreased TIA-1 expression during cellular senescence. Consistent with the previous reports showing reduced TIA-1 expression during cellular senescence in WI-38 cells and an age-related decline in TIA-1 levels across various tissues [25, 26], our in silico analysis of multiple gene expression data series (GSE) (GSE38718, GSE181022, and GSE58915) from the NCBI Gene Expression Omnibus (GEO) revealed the downregulation of *TIA-1* in aging and cellular senescence models (Fig. 2A). Additional analysis employing SeneQuest (http://senequest.org) also confirmed *TIA-1* as a gene downregulated during cellular senescence (Supplementary Fig. S2).

To further validate the decrease in TIA-1 expression during cellular senescence, we investigated relative TIA-1 expression in cellular senescence models, including replicative senescence and stress-induced senescence. Both *TIA-1* mRNA and protein levels were gradually reduced by the sequential passaging of HS27 cells, indicating a decline in TIA-1 expression during replicative senescence (Fig. 2B, C). MFF, a downstream target of TIA-1, also decreased, while cell cycle inhibitors, p27 and p21, increased in senescent cells (PDL 31 of HS27 cells) compared to young cells (PDL21 of HS27 cells) (Fig. 2C). IR exposure also suppressed TIA-1 expression in HaCaT and WI-38 cells (Fig. 2D, E, and S1), suggesting that TIA-1 expression is downregulated during stress-induced senescence. To further validate whether cellular senescence and TIA-1 reduction are concurrently occurred within individual cells, immunofluorescence assays using antibodies against γH2AX, p16^INK4a^, and TIA-1 were conducted. The results revealed that cells have γH2AX-posistive foci or p16^INK4a^ after IR exposure concurrently exhibited lower TIA-1 expression relative to their counterparts lacking such foci in HS27 and HaCaT cell lines (Fig. 2F, G). Collectively, these results indicate senescent cells have lower expression of TIA-1.

### TIA-1 knockdown promotes IR-induced cellular senescence

**Fig. 3:**
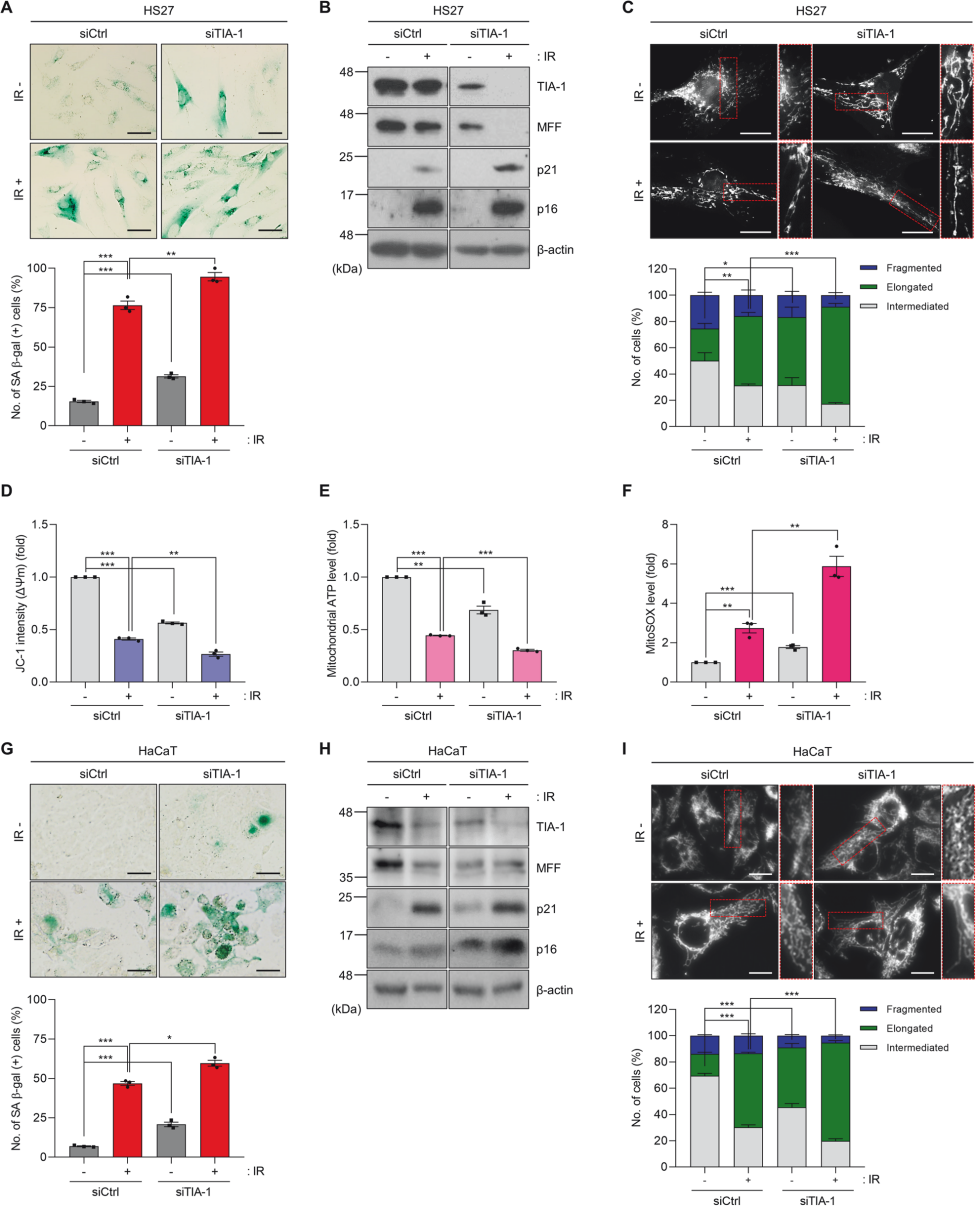
TIA-1 knockdown enhances IR-induced senescence and mitochondrial dysfunction. SA β-gal analysis (**A**), protein expression (**B**), mitochondrial morphology (**C**), and mitochondrial function (**D**–**F**) in HS27 cells. SA β-gal analysis (**G**), protein expression (**H**), and mitochondrial morphology (**I**) in HaCaT cells. Cells were transfected with siRNAs (siCtrl or siTIA-1) and exposed to IR (6 Gy, 72 h). For SA β-gal analysis (**A**, **G**), cells were incubated with a staining solution (pH 6.0), and the number of SA β-gal-positive cells was counted. Relative protein expression was determined by WB analysis (**B**, **H**). β-actin was used as a loading control for WB. Mitochondrial morphology stained with MitoTracker (100 nM) was analyzed using analyzed Image J software (**C**, **I**). Mitochondrial functionality was assessed by measuring mitochondrial membrane potential (Δψm, JC-1 staining) (**D**), ATP level (Mitochondrial ToxGlo™) (**E**), and ROS generation (MitoSOX™) (**F**) in HS27 cells following IR exposure and transfection, according to the manufacturer’s instruction. Images are representative, and the data are presented as the mean ± SEM of three independent analyses. Scale bar, 20 μm. *p < 0.05, **p < 0.01, ***p < 0.001.

To assess the potential contribution of decreased TIA-1 to cellular senescence, we investigated the induction of cellular senescence by IR exposure in HS27 cells with or without TIA-1 knockdown. TIA-1 knockdown in HS27 cells increased the levels of senescence markers, including SA β-gal, p21, and p16, while decreasing the expression of MFF, a mitochondrial dynamics regulator reported as a direct target of TIA-1 [22] (Fig. 3A, B, and S3). IR exposure increased levels of SA β-gal, p21, and p16, indicating an induction of cellular senescence in HS27 cells. This effect was further augmented by TIA-1 knockdown (Fig. 3A, B). Additionally, IR exposure led to mitochondrial elongation, an effect that was intensified by TIA-1 knockdown (Fig. 3C). Moreover, the knockdown of TIA-1 amplified the adverse effects of IR exposure on mitochondrial function, including a more pronounced decreases in mitochondrial membrane potential, a greater reduction in ATP synthesis, and an increased production of reactive oxygen species (ROS), underscoring the critical role of TIA-1 in modulating mitochondrial integrity and function in response to IR exposure (Fig. 3D–F). The enhancement of cellular senescence and mitochondrial elongation by TIA-1 knockdown was also observed in HaCaT cells (Fig. 3G–I) and MEF cells (Supplementary Fig. S4) in response to IR exposure. These results indicate that TIA-1 downregulation can lead to cellular senescence.

**Fig. 4:**
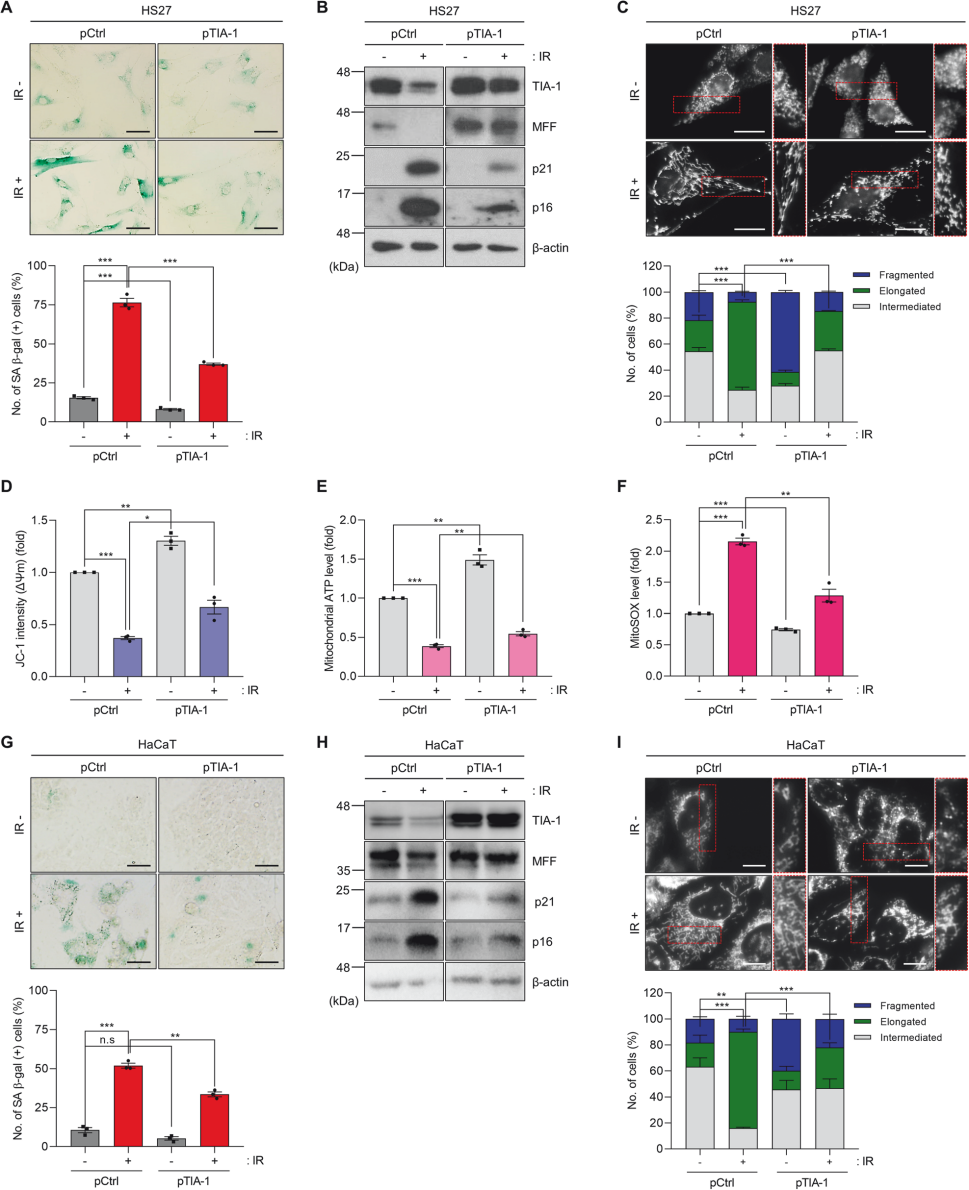
TIA-1 overexpression attenuates IR-induced senescence and mitochondrial dysfunction. SA β-gal (**A**), protein analysis (**B**), mitochondrial morphology (**C**), and mitochondrial function (**D**–**F**) in HS27 cells. SA β-gal (**G**), protein analysis (**H**), and mitochondrial morphology (**I**) in HaCaT cells. Cells were transfected with plasmids (pCtrl or pTIA-1) and exposed to IR (6 Gy, 72 h). For SA β-gal analysis (**A**, **G**), cells were incubated with a staining solution (pH 6.0), and the number of SA β-gal-positive cells was counted. Relative protein expression was determined by WB analysis (**B**, **H**). β-actin was used as a loading control for WB. Mitochondrial morphology stained with MitoTracker (100 nM) was analyzed using Image J software (**C**, **I**). Mitochondrial functionality was assessed by measuring mitochondrial membrane potential (Δψm, JC-1 staining) (**D**), ATP level (Mitochondrial ToxGlo™) (**E**), and ROS generation (MitoSOX™) (**F**) in HS27 cells following IR exposure and transfection, according to the manufacturer’s instruction. Images are representative, and the data are presented as the mean ± SEM of three independent analyses. Scale bar, 20 μm. n.s not significant (*p* > 0.05), *p < 0.05, **p < 0.01, ***p < 0.001.

To investigate the capacity of TIA-1 ectopic expression in counteracting the cellular senescence triggered by IR exposure, we examined the effects of TIA-1 overexpression on markers of cellular senescence, mitochondrial morphology, and mitochondrial function. TIA-1 overexpression resulted in a reduction of senescence markers such as SA β-gal, p21, and p16, which were upregulated by IR exposure in HS27 cells (Fig. 4A, B). Furthermore, ectopic expression of TIA-1 mitigated the adverse effects in mitochondrial dynamics and function, such as mitochondrial elongation, reduction of membrane potential and ATP synthesis, and increased ROS generation, which were triggered by IR exposure in HS27 cells (Fig. 4C–F). The anti-senescence capacity of TIA-1 was further demonstrated in HaCaT cells and MEF­/­cells following TIA-1 overexpression, as shown in Figs. 4G–I and S4, indicating the pivotal role of TIA-1 in regulating cellular senescence and mitochondrial homeostasis. These findings collectively suggest that TIA-1 has the potential to attenuate cellular senescence in response to IR exposure.

### MicroRNA-30-5p is responsible for TIA-1 downregulation during cellular senescence

**Fig. 5:**
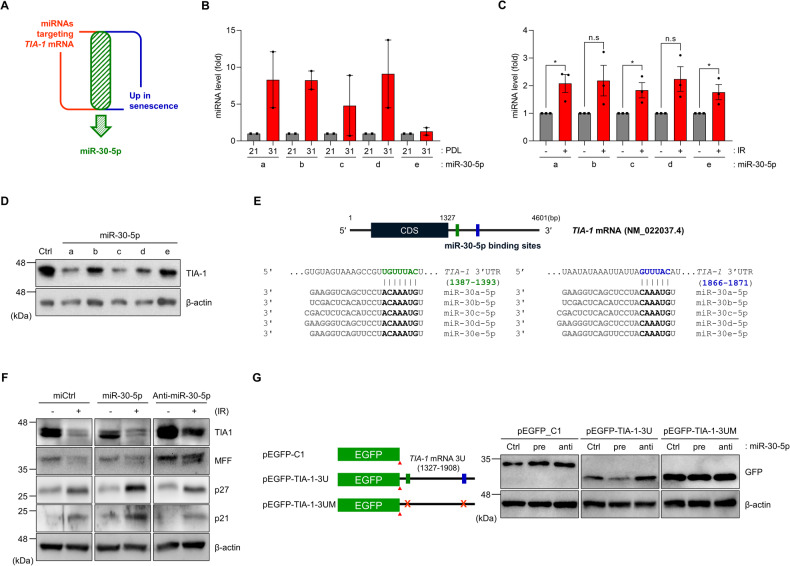
The miR-30-5p family is responsible for the downregulation of TIA-1. **A** Identification of the miR-30-5p family as a putative regulator of TIA-1 expression. In silico prediction with TargetScan (https://www.targetscan.org/vert_80/) and comparative analysis using senescence GSE datasets (GSE14912, GSE22134, GSE48662, and GSE90942) identified the miR-30-5p family. **B** MiR-30-5p family levels in young and senescent HS27 cells. **C** MiR-30-5p family levels in HaCaT cells with or without IR exposure (6 Gy, 72 h). Relative levels of each miRNA were determined by RT-qPCR analysis. *U6* snRNA was used as the reference gene for normalization. **D** TIA-1 protein levels were assessed by WB analysis after transfection with miRNAs (48 h). **E** A schematic diagram of miR-30-5p binding sites on the *TIA-1* mRNA 3′UTR. Each identical binding site is represented as a colored rectangle. **F**, **G** WB analysis. HS27 cells were transfected with miRNAs (miCtrl, precursor of miR-30-5p, and anti-miR-30-5p) and exposed to IR (6 Gy) (**E**). HEK293T cells were sequentially transfected with miRNAs and EGFP reporter plasmids (pEGFP-C1, pEGFP-TIA-3U, and pEGFP-TIA-1-3UM) (**G**). Relative protein levels of each sample were determined by WB analysis. β-actin was used as the loading control for WB. Images are representative, and the data are presented as the mean ± SEM of two (for B) or three independent analyses. n.s not significant (*p* > 0.05), *p < 0.05.

TIA-1 expression undergoes downregulation during cellular senescence, but the specific mechanisms driving TIA-1 reduction remain elusive. To elucidate the regulatory mechanism of TIA-1 downregulation in cellular senescence, we explored the potential involvement of microRNAs (miRNAs) as causal factors in TIA-1 decreases. Given that miRNAs function as negative regulators of gene expression and display dynamic alterations in response to various cellular conditions [27, 28], we hypothesized that miRNAs exhibiting increased levels during cellular senescence could regulate TIA-1 expression. Through in silico prediction with TargetScan (https://www.targetscan.org/vert_80/) and comparative analysis using GSE datasets from diverse senescence models (GSE14912, GSE22134, GSE48662, and GSE90942), we identified the miR-30-5p family as a prime candidate among miRNAs that could target TIA-1, while concurrently displaying increased expression during cellular senescence (Figs. 5A and S5). We experimentally verified the relative abundance of each member of the miR-30-5p family using RT-qPCR in models of cellular senescence. While each miR-30-5p family member exhibited distinct fold changes, all of them demonstrated augmented expression during replicative senescence (Fig. 5B) and IR-induced senescence (Fig. 5C).

To ascertain the potential role of the miR-30-5p family in downregulating TIA-1 expression, we transfected cells with the miRNA precursors for each member and performed Western blotting analysis to assess TIA-1 levels. Figure 5D demonstrates that miR-30a-5p, -30c-5p, and -30d-5p decreased TIA-1 expression, whereas the impact of miR-30b-5p and -30e-5p on TIA-1 downregulation was moderate.

Notably, the miR-30-5p family has conserved seed regions across its members, along with two binding sites on *TIA-1* mRNA (specifically at positions 1387–1393 nt and 1866–1871 nt of the 3′UTR of *TIA-1* mRNA, NM_022307) (Fig. 5E). To further validate the miR-30-5p-mediated regulation of TIA-1, we examined the relative expressions of endogenous TIA-1 and EGFP reporters upon miR-30-5p overexpression. Our findings indicated that the ectopic expression of miR-30-5p decreased TIA-1 expression, whereas the inhibition of miR-30-5p increased it (Fig. 5F). In addition, miR-30-5p downregulated EGFP reporter levels containing the 1327–1908 nt region of the *TIA-1* mRNA 3′UTR (pEGFP-TIA-1-3U) but had no effect on the expression of a mutant reporter lacking miR-30-5p binding sites (pEGFP-TIA-1-3UM) (Fig. 5G). Moreover, the inhibition of miR-30-5p using anti-miR-30-5p led to a reversal of EGFP reporter expression. Collectively, these results suggest that miR-30-5p is upregulated in senescent cells and contributes to decrease in TIA-1 expression.

### miR-30-5p promotes cellular senescence and mitochondrial elongation

**Fig. 6:**
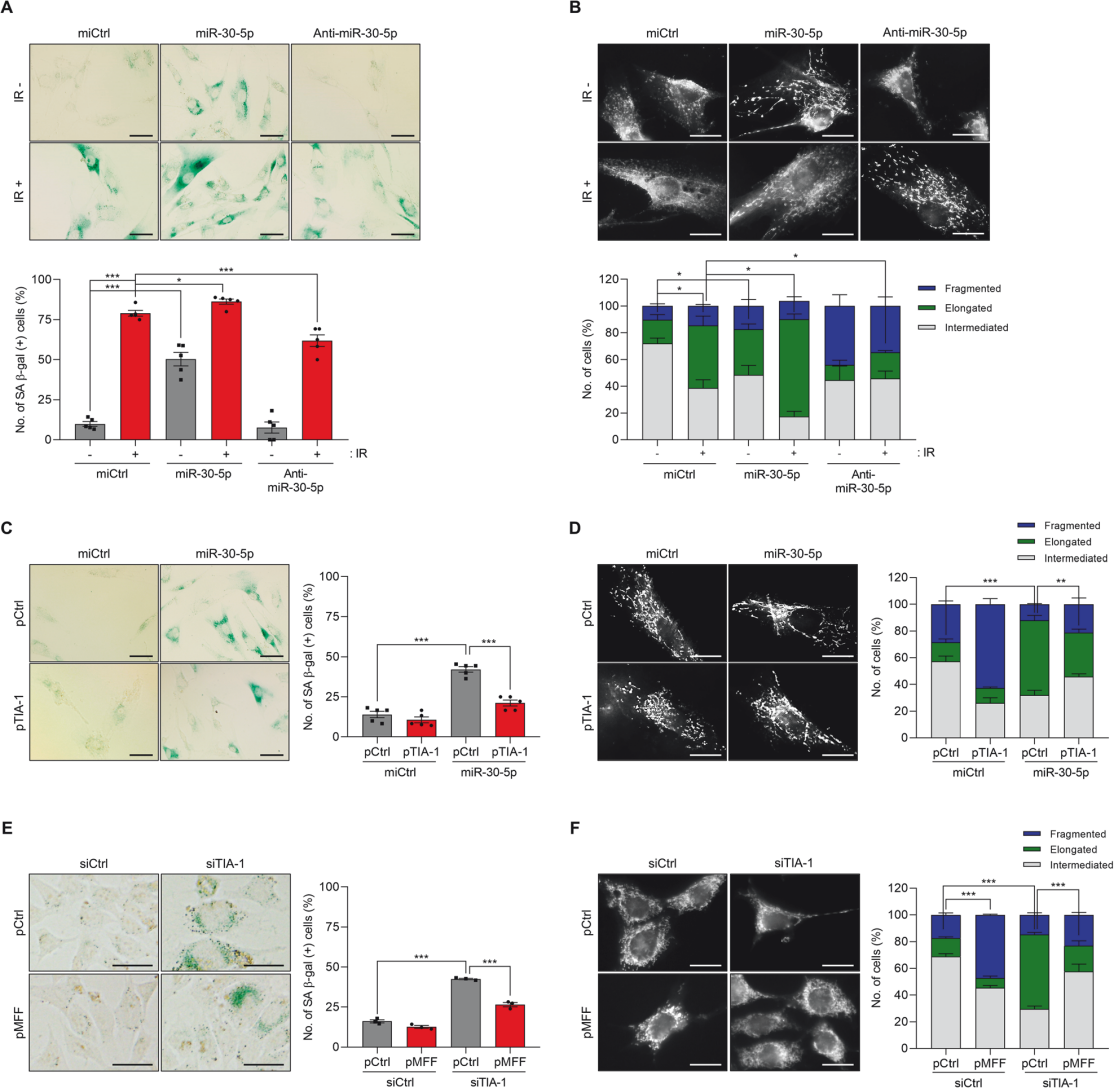
The miR-30-5p/TIA-1/MFF axis regulates cellular senescence and mitochondrial elongation. **A**, **B** SA β-gal analysis and mitochondrial morphology in HS27 cells transfected with miRNAs (miCtrl, precursor of miR-30-5p, and anti-miR-30-5p). After transfection, the cells were exposed to IR (6 Gy, 72 h). **C**, **D** SA β-gal analysis and mitochondrial morphology in HS27 cells sequentially transfected with miRNAs (miCtrl and precursor of miR-30-5p) and plasmids (pCtrl and pTIA-1). **E**, **F** SA β-gal analysis and mitochondrial morphology in HaCaT cells sequentially transfected with siRNAs (siCtrl and siTIA-1) and plasmids (pCtrl and pMFF). For SA β-gal analysis, cells were incubated with a staining solution (pH 6.0), and the number of SA β-gal-positive cells was counted. Mitochondrial morphology stained with MitoTracker (100 nM) was analyzed using Image J software. Images are representative, and the data are presented as the mean ± SEM of three independent analyses. Scale bar, 20 μm. *p < 0.05, **p < 0.01, ***p < 0.001.

To investigate the potential role of miR-30-5p in regulating cellular senescence and mitochondrial elongation, we examined the relative SA β-gal expression in HS27 cells following IR exposure, after transfection with precursor miR-30-5p or anti-miR-30-5p. Transfection with miR-30-5p intensified SA β-gal expression in response to IR exposure, whereas the inhibition of miR-30-5p led to its reduction (Fig. 6A). Moreover, the presence of miR-30-5p augmented the population of HS27 cells exhibiting elongated mitochondria, a phenomenon further accentuated by IR exposure-induced mitochondrial elongation (Fig. 6B). However, anti-miR-30-5p failed to stimulate IR-induced mitochondria elongation in HS27 cells. The effects of miR-30-5p on cellular senescence, mitochondrial elongation, and TIA-1 regulation were also reproducible in MCF7 (Supplementary Fig. S6). These observations collectively indicate that miR-30-5p has the potential to promote cellular senescence and mitochondria elongation.

Furthermore, to ascertain whether TIA-1 overexpression could counteract miR-30-5p-induced cellular senescence and mitochondrial elongation, we conducted sequential overexpression of miR-30-5p and TIA-1 in HS27 cells and subsequently evaluated SA β-gal expression and mitochondrial length. The introduction of TIA-1 through ectopic expression alleviated cellular senescence and mitochondrial elongation induced by miR-30-5p overexpression, which indicates that miR-30-5p induces cellular senescence and changes in mitochondrial dynamics through the downregulation of TIA-1 (Fig. 6C, D). To further elucidation of downstream mechanism of TIA-1 action, we explored the relevance of MFF, a downstream target of TIA-1. Ectopic expression of MFF counteracted the mitochondrial elongation and cellular senescence induced by TIA-1 knockdown (Fig. 6E, F). Collectively, these results suggest that the miR-30-5p/TIA-1/MFF axis plays an important role in regulating cellular senescence and mitochondrial dynamics.

## Discussion

The intricate relationship between cellular senescence and mitochondrial dysfunction has been extensively investigated. Prevailing research demonstrated that senescent cells exhibited heightened mitochondrial mass, an augmented generation of ROS, perturbed mitochondrial membrane potential, and irregular mitochondrial morphology, all contributing to mitochondrial dysfunction. However, recent studies proposed that mitochondrial dysfunction might not solely be a passive outcome of cellular senescence, as it appears to actively promote the process [29–33]. Dysregulated factors, such as excessive ROS production, altered NAD+ metabolism, mitochondrial calcium accumulation, and impaired mitochondrial dynamics have been linked to accelerated cellular senescence and age-associated traits across diverse models [34].

In this study, we presented the hyperfusion of mitochondria and decreases in TIA-1 expression in cellular senescence. TIA-1 knockdown resulted in an elevation in the expression of multiple senescence markers and an increase in mitochondrial length across various cell types. The introduction of TIA-1 via ectopic expression mitigated cellular senescence by decreasing mitochondrial hyperfusion under stress conditions, such as IR exposure. Furthermore, we identified the miR-30-5p family as a causal factor in TIA-1 downregulation during cellular senescence. The miR-30-5p family level was upregulated in senescence models and the overexpression of miR-30-5p led to decreases in TIA-1 and mitochondrial fusion, thereby promoting cellular senescence. The inhibition of miR-30-5p counteracted IR-induced cellular senescence by alleviating TIA-1-mediated mitochondrial fission. Collectively, these outcomes underscore the pivotal role played by TIA-1 downregulation in promoting cellular senescence by inducing mitochondrial hyperfusion.

In-depth exploration, encompassing in silico analysis and experimental validation, consistently revealed a decrease in TIA-1 expression across various senescence models, including replicative and stress-induced senescence (Fig. 2). Our investigations pinpointed the miR-30-5p family as a regulatory factor driving TIA-1 downregulation. The ectopic expression of miR-30-5p downregulated TIA-1 levels, enhanced mitochondrial elongation, and increased the expression of SA β-gal, while the inhibition of miR-30-5p effectively reversed these phenomena. This collective evidence underscores the pivotal role played by increased miR-30-5p in the downregulation of TIA-1 during cellular senescence.

The *TIA-1* gene exists in two distinct isoforms, TIA-1a and TIA-1b, through the alternative splicing of exon 5 [35]. The exon-skipping product TIA-1b is missing 11 amino acids encoded in exon 5 of the *TIA-1* gene compared to TIA-1a. While the characteristics of each isoform in esophageal squamous cell carcinoma have been reported [36], their expression and function in other cell types are not yet known. Subsequent investigations into TIA-1 isoforms promise detailed insight into TIA-1-mediated gene expression in various cellular processes, including cellular senescence.

We proposed the pro-senescent role of the miR-30-5p family by targeting TIA-1 (Fig. 5). Consistent with our findings, a prior report employing human HeLa cells showed that the upregulation of miR-30-5p promoted cellular senescence by suppressing b-Myb expression [37]. However, a contrasting study reported that miR-30-5p disrupted Ras-induced senescence and promotes cancer development in murine models [38]. This discrepancy suggests the possibility of the differential regulation of miR-30-5p family expression between humans and mice. While this study investigated the expression of the miR-30-5p family in human cell lines, including HS27 and HaCaT, additional experiments await further analysis to elucidate the biological and clinical significance of the miR-30-5p family in cellular senescence.

Our previous work showed that TIA-1 plays an essential role in mitochondrial dynamics by regulating MFF expression [22]. Additionally, target mRNAs of TIA-1, including tumor necrosis factor α, PRKRA, cyclin A2, and OPA1 [23, 36, 39, 40], have also been identified, underscoring the pivotal function of TIA-1 as an essential factor in gene expression in various cellular processes [41]. Thus, given the observed decline in TIA-1 levels during cellular senescence (Fig. 2), it is conceivable that TIA-1-mediated gene regulation is closely involved in cellular senescence and age-related processes. Therefore, it is worth expanding our knowledge of TIA-1-mediated gene regulation within the context of cellular senescence and age-related diseases. Conducting comprehensive studies using CLIP-seq data [39, 42] may provide a chance to discover additional TIA-1 targets and the detailed regulatory mechanisms.

Dysregulation of mitochondrial dynamics is increasingly recognized as a pivotal factor in the pathogenesis of various diseases, notably cancer [43, 44]. The intricate relationship between mitochondrial behavior and the efficacy of cancer treatments is significant, with evidence suggesting that changes in mitochondrial morphology and function are closely linked to how cancer cells respond to therapies [45]. Notably, anticancer agents like cisplatin and venetoclax have been documented to induce profound morphological and functional changes in mitochondria, leading to increased apoptosis and, consequently, heightened sensitivity of cancer cells to these drugs [46–49]. Similarly, radiation therapy is known to affect mitochondrial integrity [50]. High doses of ionizing radiation (IR) caused mitochondrial fragmentation and cellular senescence [51]. Conversely, the development of resistance to anticancer drugs or radiation in certain cancer cell populations is often marked by distinct alterations in mitochondrial dynamics [52, 53]. This suggests that mitochondrial integrity plays a crucial role in the emergence of resistance to therapy, making mitochondrial dynamics a key factor in determining cancer cell survivability under treatment stress.

Beyond the immediate effects of chemotherapy and radiation, alterations in mitochondrial dynamics are implicated in broader oncogenic processes such as metabolic reprogramming, the maintenance of redox balance, and the regulation of cell survival pathways [44, 54, 55]. This broad involvement underscores the potential of mitochondrial dynamics as a target for novel therapeutic strategies. By elucidating and targeting the molecular mechanisms underlying mitochondrial dysregulation, there is potential to overcome drug resistance and improve the efficacy of existing cancer therapies, leading to more personalized and effective approaches to cancer treatment.

Taken together, our findings underscore the significance of TIA-1 in the orchestration of cellular senescence. Our results demonstrate that decrease in TIA-1 expression, driven by elevated miR-30-5p levels and subsequent mitochondrial hyperfusion, exacerbate senescent phenotypes in cells, indicating the pivotal role of the miR-30-5p/TIA-1axis in cellular senescence. Further inquiries focused on deciphering the clinical implications of TIA-1-mediated gene regulation have the potential to deepen our understanding of intricate molecular mechanisms governing cellular senescence and age-related phenomena. This endeavor may provide innovative strategies aimed at the prevention and treatment of age-related diseases.

## Materials and methods

### Cell culture and transfections

HS27 (human foreskin fibroblasts), HaCaT (human skin keratinocytes), MEF (murine embryonic fibroblast), WI-38 (human lung fibroblast), MCF7 (human breast adenocarcinoma), and HEK293T cells were cultured in Dulbecco’s modified Eagle’s medium (DMEM) (Capricorn Scientific, Ebsdorfergrund, Germany) supplemented with 10% fetal bovine serum (FBS) and 1% antibiotics at 37 °C. MEF wildtype (WT) (TIA-1 +/+) and TIA-1­ −/− ­cells were gifts from Dr. Paul Anderson (Harvard Medical School, Boston, MA, USA). Plasmids overexpressing TIA-1 and MFF were generated by inserting ORF of each gene into pCMV6-AN-His-HA mammalian expression vector using specific primer set (Supplementary Table S1). The transfection of small interfering RNAs (siRNAs) (Genolution Pharmaceuticals, Inc., Seoul, South Korea), microRNAs (BIONEER, Daejeon, South Korea), and plasmids was conducted using Lipofectamine^TM^ 2000 (Invitrogen^TM^, Waltham, MA, USA), according to the manufacturer’s instructions.

To induce replicative senescence, HS27 cells were passaged until they became senescent. HS27 cells at PDL21 were used as young cells and HS27 cells at PDL31 were used as senescent cells. To induce stress-induced cellular senescence, cells were exposed to γ-irradiation (6 Gy) using a γ-irradiator, Gammacell 3000 Elan (MDS Nordion, Ottawa, ON, Canada) and further incubated for 3 days.

### RNA analysis

Total RNA was isolated from whole cells using RNAiso Plus (Takara Bio, Inc., Shiga, Japan). cDNA was synthesized via reverse transcription using the ReverTra^®^ Ace qPCR RT kit (Toyobo Co., Ltd, Osaka, Japan) or the Mir-X^TM^ miRNA Fist-Strand Synthesis kit (Takara Bio USA, Inc., San Jose, CA, USA). Relative levels of RNAs were measured by quantitative PCR (qPCR) using the SensiFAST™ SYBR Hi-ROX kit (Meridian Bioscience, Inc., Cincinnati, OH, USA), gene-specific primers (Supplementary Table S1), and the StepOnePlus™ Real-Time PCR System (Applied Biosystems™, Waltham, MA, USA). Data were processed using the _ΔΔ_CT method for comparison between control and experimental groups. *GAPDH* mRNA (for mRNA quantification) and *U6* snRNA (for miRNA quantification) were used as reference genes for the normalization.

### Protein analysis

Whole-cell lysates were prepared using RIPA buffer containing 1× protease inhibitor cocktail (Roche, Basel, Switzerland), separated by sodium dodecyl-polyacrylamide gel electrophoresis (SDS-PAGE), and transferred onto polyvinylidene difluoride membranes (Millipore, Burlington, MA, USA). After incubating the membranes with primary antibodies against TIA-1, GFP (Santa Cruz Biotechnology, Inc., Dallas, TX, USA), MFF, MFN1, MFN2 (Abcam, Plc., Cambridge, UK), p27, p21 (Cell Signaling Technology, Inc., Danvers, MA, USA), p16, OPA1, DRP1 (BD Bioscience, Franklin Lakes, NJ, USA) or β-actin (Genetex, Inc., Irvine, CA, USA) at 4 °C overnight, they were incubated with horseradish peroxidase-conjugated secondary antibodies (Sigma-Aldrich, Burlington, MA, USA) at room temperature for 1 h. Chemiluminescence was detected by adding a Clarity Western ECL Substrate (Bio-Rad, Inc., Hercules, CA, USA) to the membrane and images were captured using the ChemiDoc Imaging Systems (Bio-Rad, Inc.).

### Microscopy and analysis of mitochondrial morphology

For fluorescence microscopy, cells were fixed, permeabilized, sequentially incubated with primary antibodies, including TIA-1 (Abcam, Plc., Cambridge, UK), γH2AX (Ser139) (Merck, Taufkirchen, Germany), and p16^INK4a^ (Santa Cruz Biotechnology, Inc., Dallas, TX, USA), and further incubated with secondary antibodies conjugated with Alexa Flour® 488 or Alexa Flour® 555 (Abcam Plc.) [56]. DAPI solution (Invitrogen™) was used to stain the nuclei. Fluorescent signals were observed using the Observer.Z1 inverted microscope (Carl Zeiss, Oberkochen, Germany). For the staining of mitochondria, cells were incubated with 100 nM of MitoTracker Red CMXRos (Invitrogen^TM^, Waltham, MA, USA) in serum-free media for 30 min, and mitochondrial morphology was analyzed by tracing fluorescent signals using the Observer.Z1 microscope and the Image J software. Cells were classified into three groups based on relative mitochondrial length: elongated, intermediated, and fragmented. The ratio of cells in each category was calculated by dividing the number of cells in the category by the total number of cells.

For transmission electron microscopy (TEM) analysis of mitochondria, cells were fixed with 1% osmium tetroxide and embedded in Epon 812. Ultrathin sections were analyzed with a transmission electron microscope (JEM 1010, Tokyo, Japan) [22]. Perimeter of mitochondria in TEM image of each experimental group was analyzed Image J software [57, 58].

### Functional analysis of mitochondria

Mitochondrial membrane potential (Δψm) and mitochondrial ATP synthesis were assessed using the JC-1 Mitochondrial Membrane Potential Assay Kit (Abcam, Plc., Cambridge, UK) and the Mitochondrial ToxGlo™ assay (Promega, Madison, WI, USA), according to the manufacturers’ instruction [22]. The fluorescence of each experimental group was measured at 535 nm (excitation)/590 nm (emission) for JC-1 staining and at 485 nm (excitation)/530 nm (emission) for the Mitochondrial ToxGlo™ assay using a Synergy H1 fluorescent plate reader (Bio-Tek, Winooski, Vermont, USA.).

For the analysis of ROS level, cells were incubated with with 2.5 μM MitoSOX™ (Invitrogen, Waltham, MA, USA) in HBSS buffer for 15 min and the fluorescence was analyzed using Observer.Z1 inverted microscope (Carl Zeiss, Oberkochen, Germany) and Image J software.

### Senescence-associated β-galactosidase assay

Cells were fixed in 4% formaldehyde (FA) and incubated in a mixture containing 40 mM citric acid/sodium phosphate (pH 6.0), 150 mM NaCl, 2 mM MgCl_2_, 5 mM potassium ferricyanide and potassium ferrocyanide, and 1 mg/mL X-gal (BEAMS Biotechnology, Seongnam, South Korea), at 37 °C for 16 h in dark [59]. After washing with 1× phosphate-buffered saline (PBS), β-gal signals were observed using an IX70w microscope (Olympus Corp., Tokyo, Japan). The number of β-gal-positive cells was determined by counting cells in the images obtained from three independent experiments. The ratio of senescent cells was calculated by dividing the number of β-gal-positive cells by the total number of cells.

### EGFP reporter analysis

The reporter plasmid containing miR-30-5p binding sites (pEGFP-TIA-1 3U) was generated by inserting a 3’UTR fragment of *TIA-1* mRNA (NM_022037, 1327–1908, 582 nt) into pEGFP-C1 (BD Bioscience). The mutant reporter plasmid (pEGFP-TIA-1 3UM) was generated by site-directed mutagenesis using the KOD plus mutagenesis kit (Toyobo, Japan) and a specific primer set (Supplementary Table S1). After transfection with the miR-30-5p precursor or anti-miR-30-5p, the cells were sequentially transfected with reporter plasmids for 24 h and the relative expression of the EGFP reporter was assessed by Western blotting analysis.

### In silico analysis of the GSE dataset

Gene expression profiling data sets were obtained from the National Center of Biotechnology Information (NCBI) Gene Expression Omnibus (GEO) database. GSE14912, GSE22134, GSE48662, and GSE90942 sets were used to analyze the miR-30-5p family levels. GSE38718, GSE181022, and GSE58915 sets were used to analyze *TIA-1* mRNA.

### Statistical analysis

Data are expressed as the mean ± SEM of three independent experiments. The statistical significance of the data was analyzed by the Student’s *t*-test (n.s., not significant with *p* > 0.05; *p < 0.05; **p < 0.01; ***p < 0.001).

### Supplementary information

Supplementary materials

Original Data File

## Data Availability

The data analyzed during this study are included in this published article and the supplemental data files.
