# Supplementary material for: The miR-30-5p/TIA-1 axis directs cellular senescence by regulating mitochondrial dynamics
Source: Cell Death Dis. 2024 Jun 10;15(6):404. doi: 10.1038/s41419-024-06797-1 (PMC11164864; doi:10.1038/s41419-024-06797-1)
Supplement: Supplementary file 1 — Supplementary materials [file 41419_2024_6797_MOESM1_ESM.docx]

**The miR-30-5p/TIA-1 axis directs cellular senescence by regulating mitochondrial dynamics**

​Hyosun Tak^1, 2, $^, Seongho Cha^1, 3, $^, Youlim Hong^1^, Myeongwoo Jung^1,4^, Seungyeon Ryu^1,3^, Sukyoung Han^1, 3^, Seung Min Jeong^1, 3, 4^, Wook Kim^5^, and Eun Kyung Lee^1, 3, 4, *^

^1^ Department of Biochemistry, The Catholic University of Korea, Seoul 06591, South Korea

^2^ INSERM U1052, CNRS UMR-5286, Cancer Research Center of Lyon (CRCL), Lyon, 69008, France

^3^ Department of Biomedicine & Health Sciences, The Catholic University of Korea, Seoul 06591, South Korea

^4^ Institute for Aging and Metabolic Diseases, College of Medicine, The Catholic University of Korea, Seoul 06591, South Korea

^5^ Department of Molecular Science & Technology, Ajou University, Suwon 16499, South Korea

^$^ These authors contribute equally.

^*^ Correspondence should be addressed to Eun Kyung Lee; [leeek@catholic.ac.kr](mailto:leeek@catholic.ac.kr)


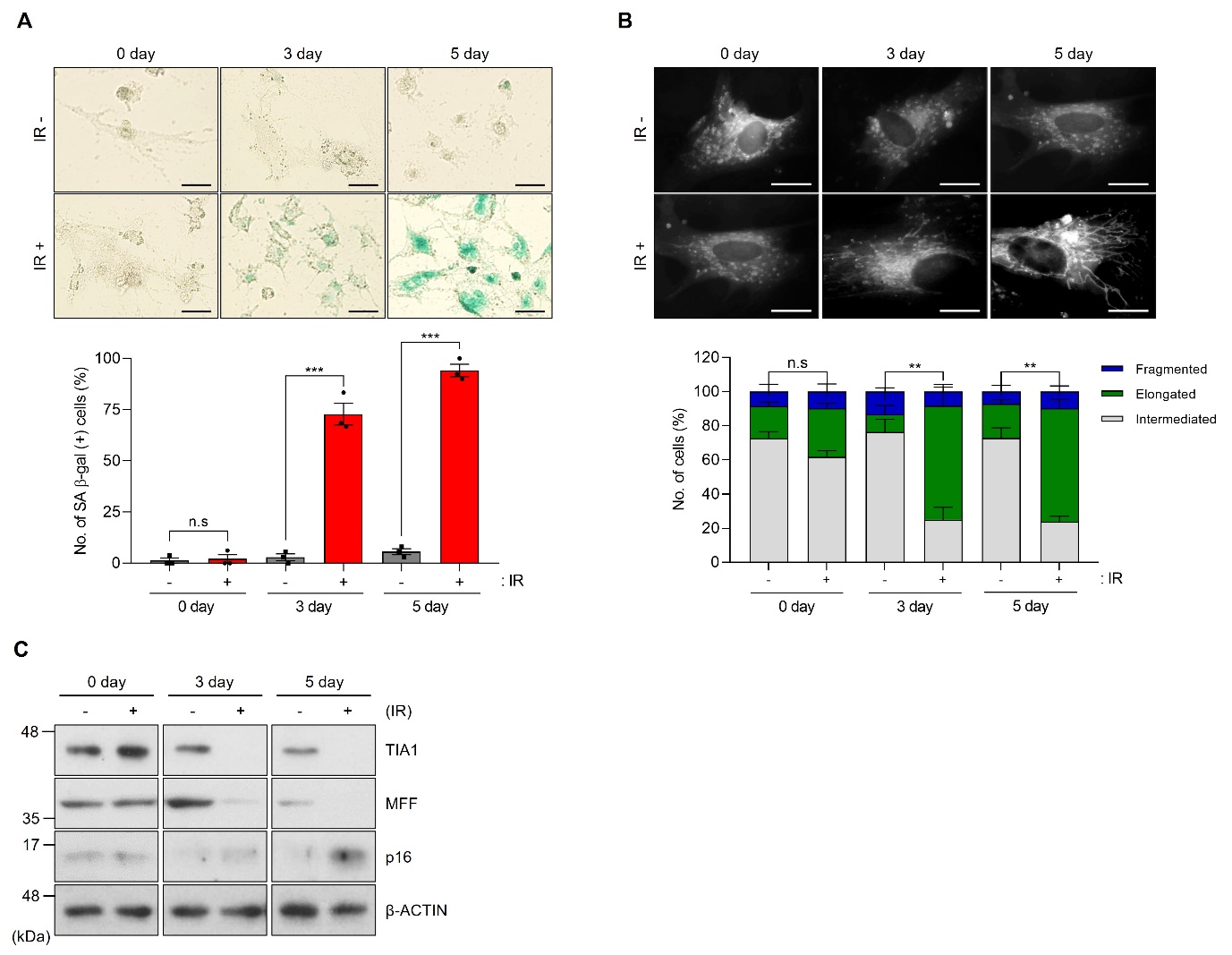


**Supplementary Figure S1. IR exposure induces cellular senescence and mitochondrial elongation in WI-38 human lung fibroblasts.**

(A) SA β-gal analysis, (B) mitochondrial morphology, and (C) protein expression in WI-38 cells. Cells were exposed to IR (6 Gy) and further incubated for the indicated time. For SA β-gal analysis, cells were incubated with a staining solution (pH 6.0), and the number of SA β-gal-positive cells was counted. Mitochondrial morphology was assessed by staining with Mitotracker (100 nM). The number of cells with fragmented, elongated, or intermediated mitochondria was counted. Protein expression was determined by WB analysis. β-actin was used as the loading control for WB. Images are representative, and the data are presented as the mean ± SEM of three independent analyses. Scale bar, 20 μm. n.s, not significant (*p* > 0.05), ***p* < 0.01, ****p* < 0.001.


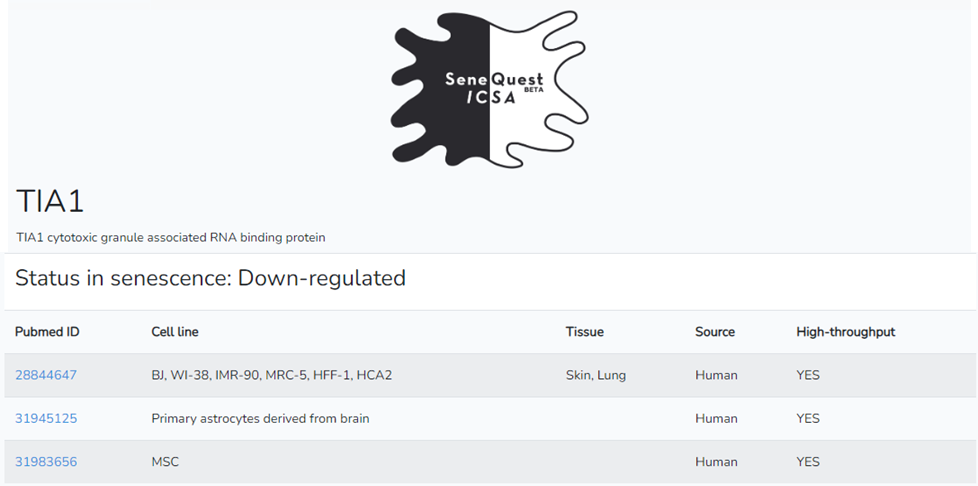


**Supplementary Figure S2. Analysis of TIA-1 expression in cellular senescence using the SeneQuest database.**

TIA-1 expression in senescence analyzed in the SeneQuest database (https://senequest.net/).


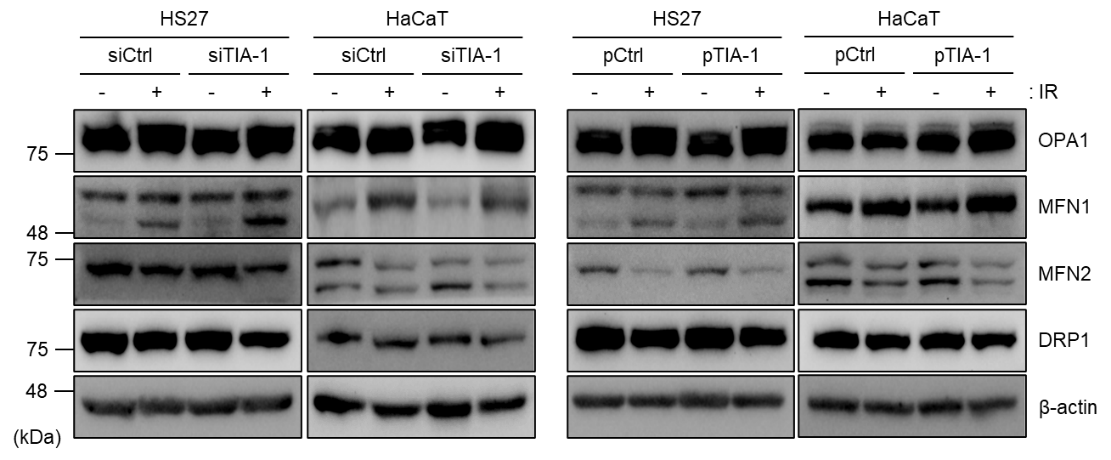


**Supplementary Figure S3. Relative expression of factors regulating mitochondrial dynamics in HS27 and HaCaT cells fowlloing IR exposure and TIA-1 regulation.**

HS27 and HaCaT cells were transfected with siRNAs (siCtrl or siTIA-1) or plasmids (pCtrl or pTIA-1) and further incubated after IR exposure (6 Gy, 72 h). Relative protein expression of each experimental group was determined by WB analysis. β-actin was used as a loading control for WB. Images are presented as the representative image of three independent analyses.

**
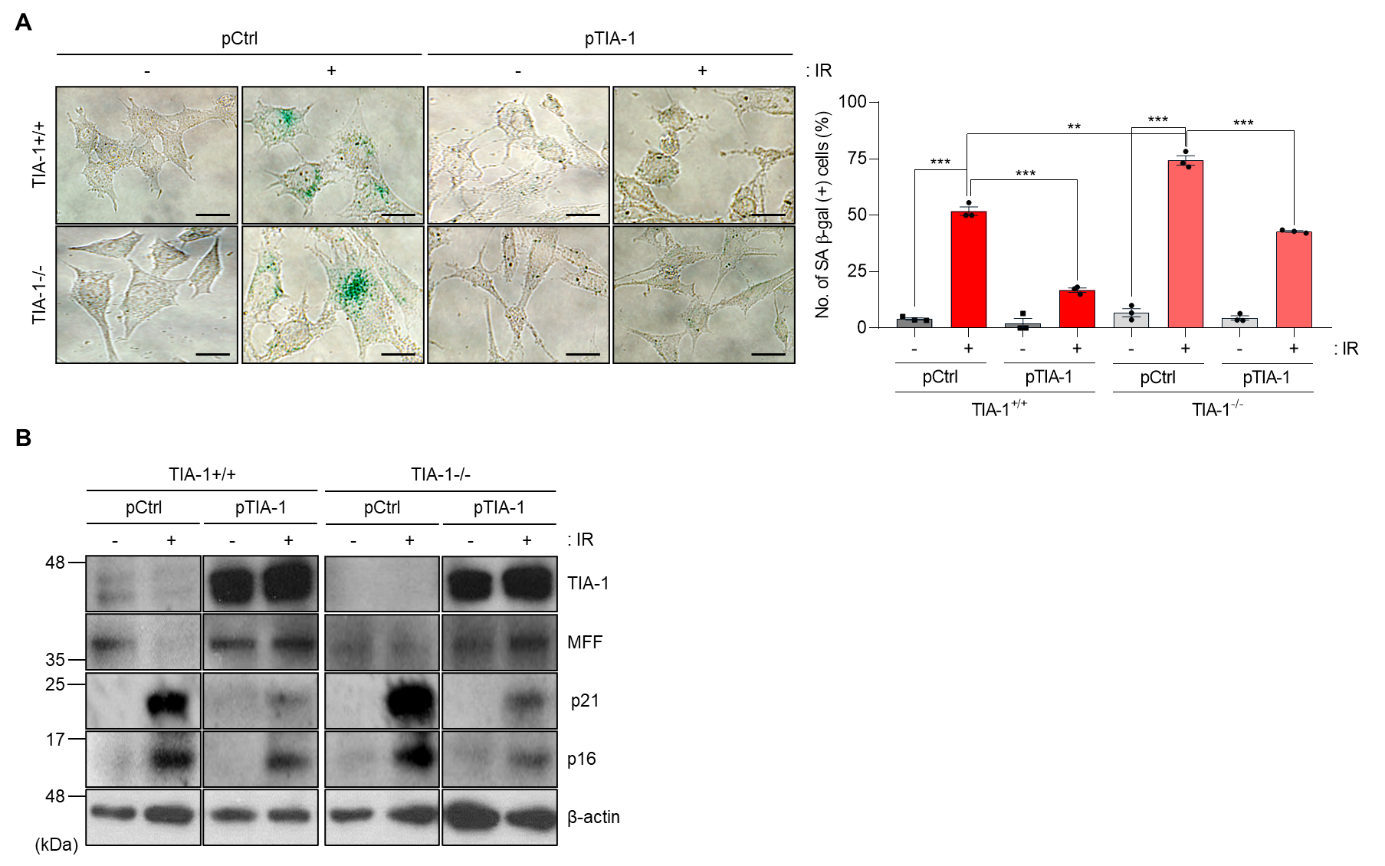
**

**Supplementary Figure S4. TIA-1 overexpression alleviates IR-induced senescence in TIA-1 -/- MEF cells.**

(A) SA β-gal analysis. (B) Protein expression in MEF cells. (A) MEF wildtype (TIA-1 +/+) and TIA-1 -/- cells were transfected with plasmids (pCtrl and pTIA-1) and exposed to IR (6 Gy). SA β-gal and protein levels were analyzed after 72 h of incubation. For SA β-gal analysis, cells were incubated with a staining solution (pH 6.0), and the number of SA β-gal-positive cells was counted. Protein expression was determined by WB analysis. β-actin was used as the loading control for WB. Images are representative, and the data are presented as the mean ± SEM of three independent analyses. Scale bar, 20 μm. ***p* < 0.01, ****p* < 0.001.

**
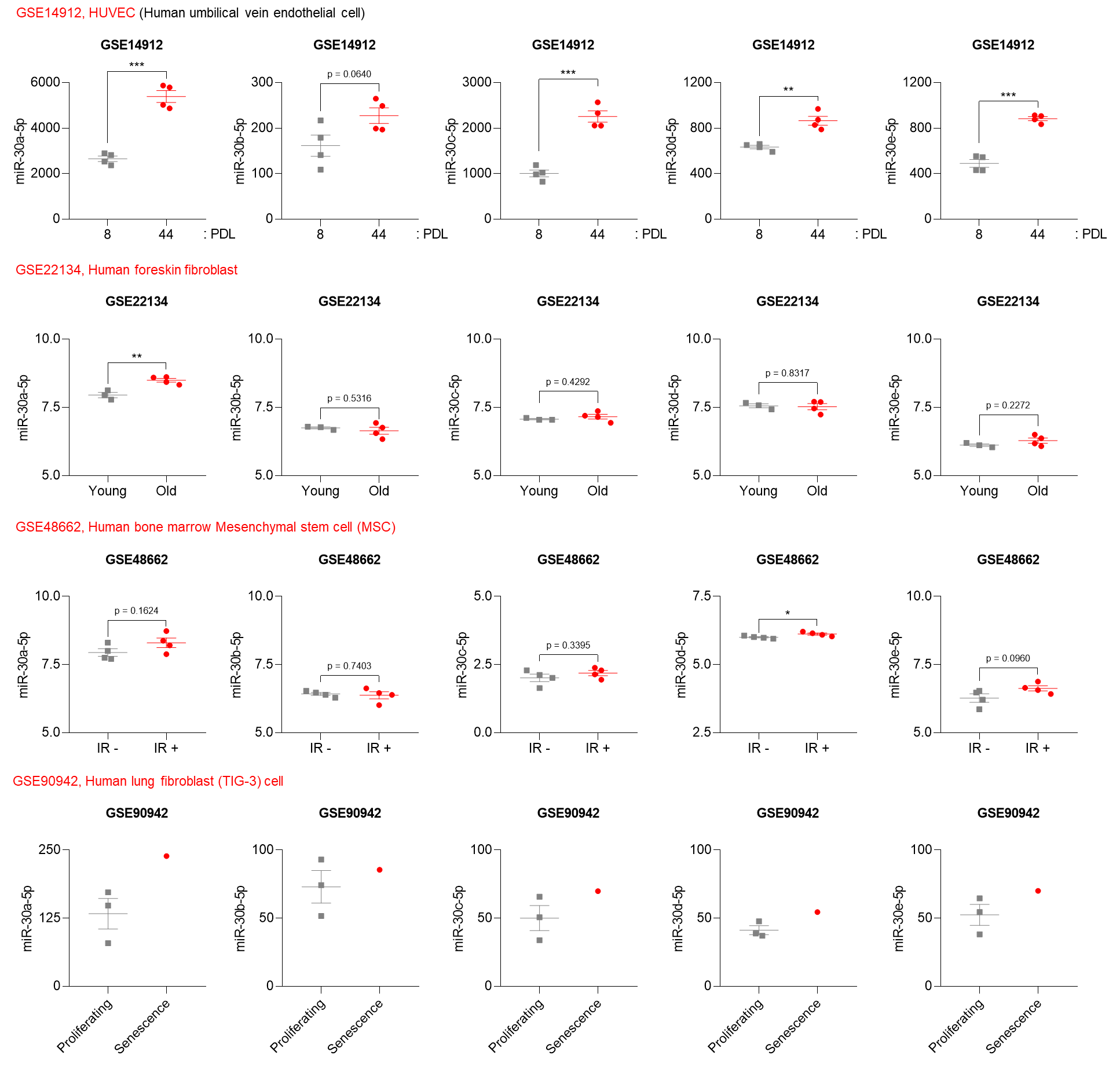
**

**Supplementary Figure S5. Relative expression of the miR-30-5p family in multiple senescent transcriptomes.**

Expression profiles of the miR-30-5p family in multiple gene expression data series (GSE), including GSE14912, GSE22134, GSE48662, and GSE90942.


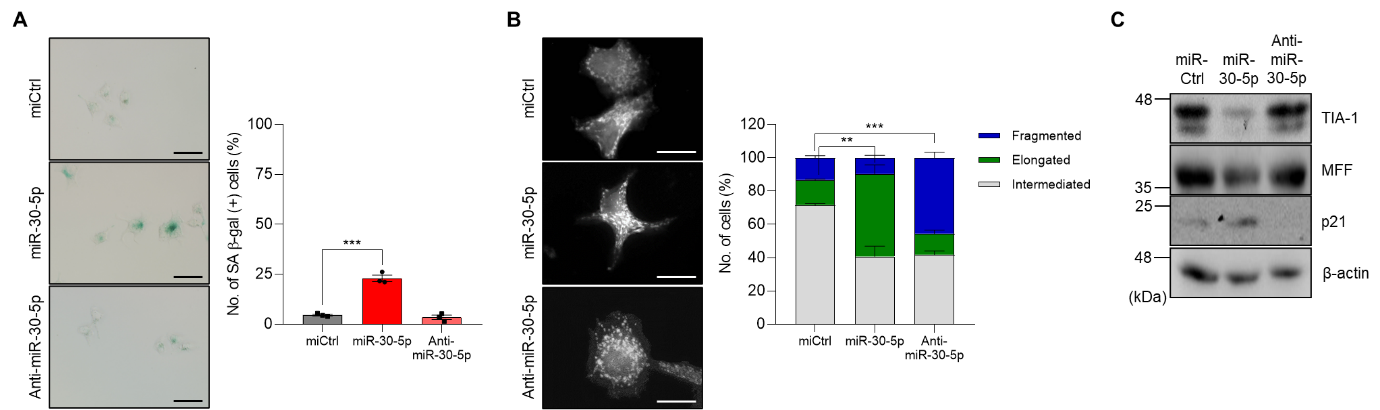


**Supplementary Figure S6. miR-30-5p induces cellular senescence and mitochondrial elongation by targeting TIA-1.**

(A) SA β-gal analysis, (B) mitochondrial morphology, and (C) protein expression in MCF7 human breast cancer cells. Cells were transfected with miRNAs (miCtrl, precursor of miR-30-5p, and anti-miR-30-5p) for 48 h, and relative SA β-gal expression, mitochondrial morphology, and protein levels were determined by SA β-gal staining, Mitotracker staining, and WB analysis, respectively. β-actin was used as the loading control for WB. Images are representative, and the data are presented as the mean ± SEM of three independent analyses. Scale bar, 20 μm. The statistical significance of data was analyzed by the Student’s t-test: ***p* < 0.01, ****p* < 0.001.

**Supplementary Table S1. Oligonucleotide sequences used in this study**

| **For RT-qPCR** | **Forward primers (5ʹ → 3ʹ)** | **Reverse primers (5ʹ → 3ʹ)** |
| --- | --- | --- |
| *TIA-1* | CGAGATGCCCAAGACTCTATACG | CCTTACCCATTATCTTCCGTCCA |
| *GAPDH* | AGGTCGGTGTGAACGGATTTG | TGTAGACCATGTAGTTGAGGTCA |
| miR-30a | TGTAAACATCCTCGACTGGAAG | mRQ 3′ Primer  (Mir-XTM miRNA Fist-Strand Synthesis kit, Takara Bio USA, Inc., Cat #. 638313) |
| miR-30b | TGTAAACATCCTACACTCAGCT |  |
| miR-30c | TGTAAACATCCTACACTCTCAGC |  |
| miR-30d | TGTAAACATCCCCGACTGGAAG |  |
| miR-30e | TGTAAACATCCTTGACTGGAAG |  |
| U6 snRNA | U6 Forward primer  (Mir-XTM miRNA Fist-Strand Synthesis kit, Takara Bio USA, Inc., Cat #. 638313) | U6 Reverse primer  (Mir-XTM miRNA Fist-Strand Synthesis kit, Takara Bio USA, Inc., Cat #. 638313) |
| **For cloning** | **Forward primers (5ʹ → 3ʹ)** | **Reverse primers (5ʹ → 3ʹ)** |
| pEGFP-TIA-1-3U | AAAAAGATCTTAAATAAGGACTCCA | AAAAGGTACCACAAATTTGTGAAAAAA |
| pEGFP-TIA-1-3MU #1 | GTGTAGTAAAGCCGTCCCCCACTTAAAGATTTATCA | TGATAAATCTTTAAGTGGGGGACGGCTTTACTACAC |
| pEGFP-TIA-1-3MU #2  pMFF  pTIA-1 | TAATATAAATTATTACCCCCCATCATTTTTGTATCT  AAAAGCGATCGCCATGAGTAAAGGAACAAGC  AAAAGCGATCGCCATGGAGGACGAGATGCCC | AGATACAAAAATGATGGGGGGTAATAATTTATATTA  AAAACTCGAGCTAGCGGCGAAACCAGAG  AAAACTCGAGTCACTGGGTTTCATACCC |
| **siRNA** | **Sense sequences (5ʹ → 3ʹ)** | **Antisense sequences (5ʹ → 3ʹ)** |
| Control siRNA (siCtrl) | AAUUCUCCGAACGUGUCACGUUU | ACGUGACACGUUCGGAGAAUUU |
| siTIA-1 | AACACAACAAAUUGGCCAGUAUU | UACUGGCCAAUUUGUUGUGUU |
