# Supplementary figures and images for: The miR-30-5p/TIA-1 axis directs cellular senescence by regulating mitochondrial dynamics
Source: Cell Death Dis. 2024 Jun 10;15(6):404. doi: 10.1038/s41419-024-06797-1 (PMC11164864; doi:10.1038/s41419-024-06797-1)

Uncropped WB images

Fig. 2C

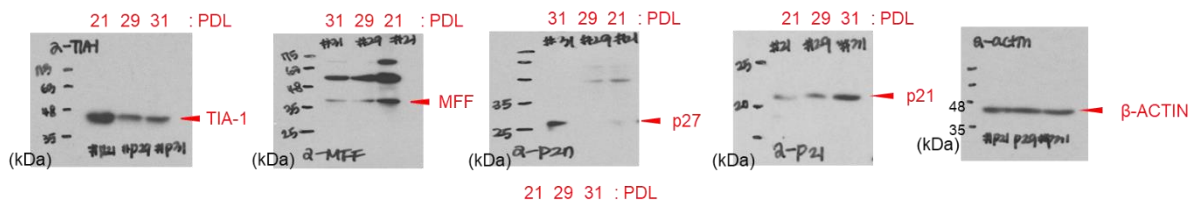

Fig. 2E

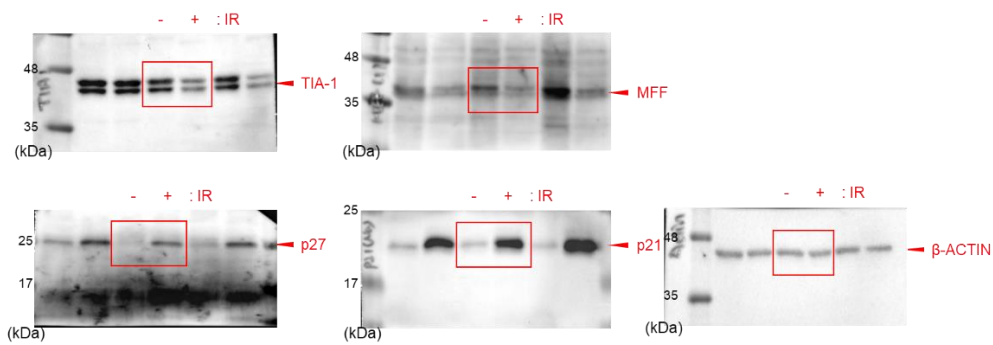

Fig. 3B

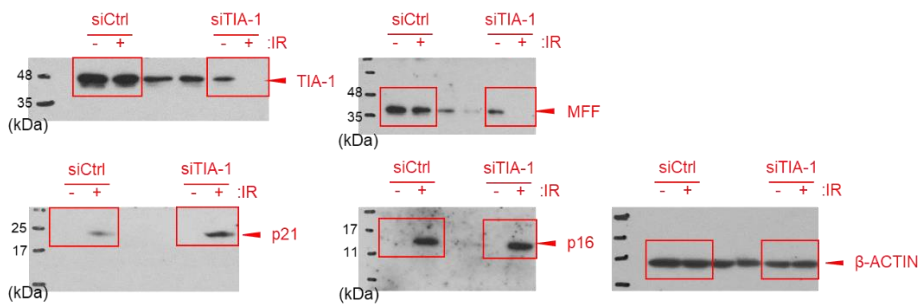

Fig. 3E

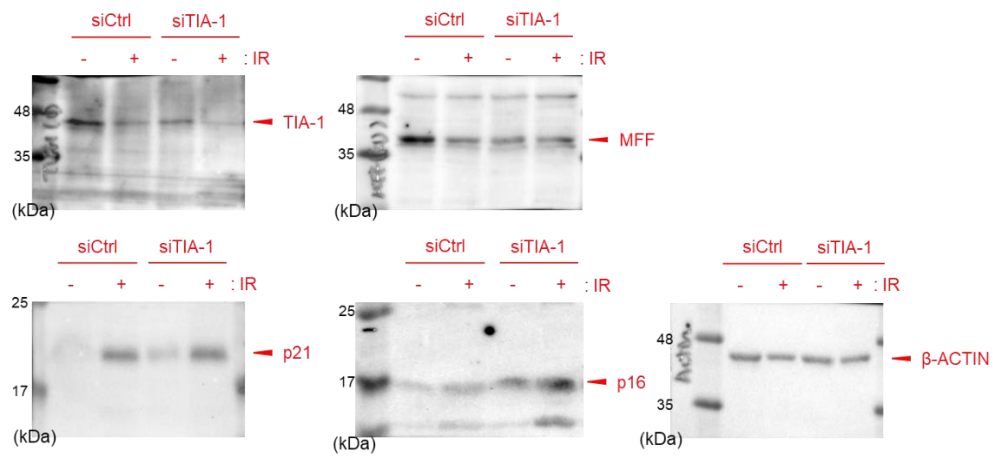

Fig. 4B

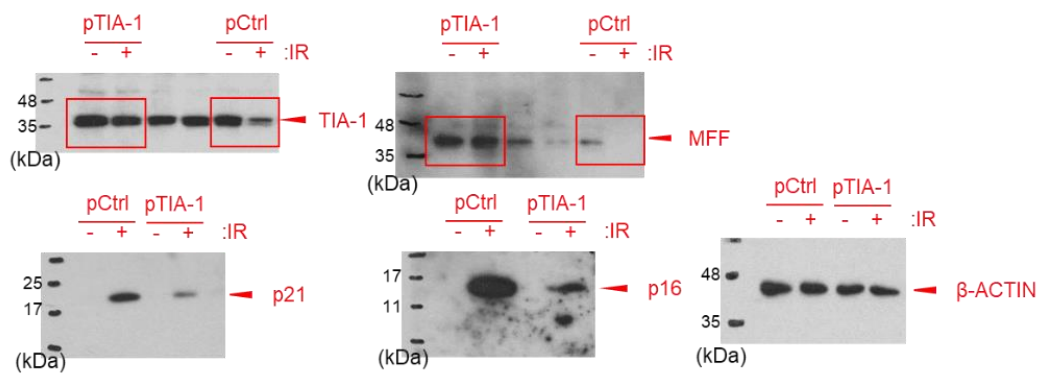

Fig. 4D

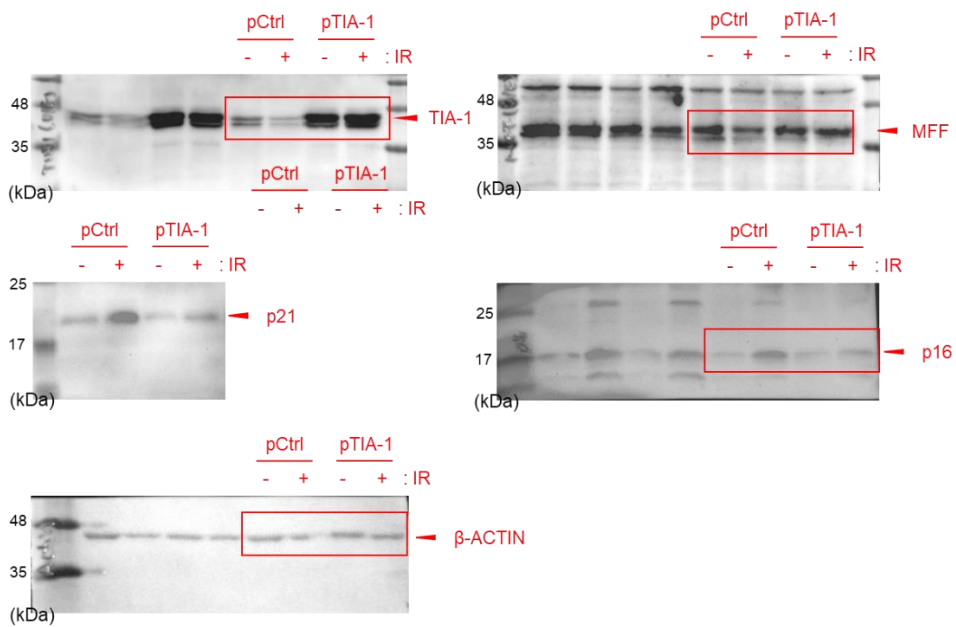

Fig. 5D

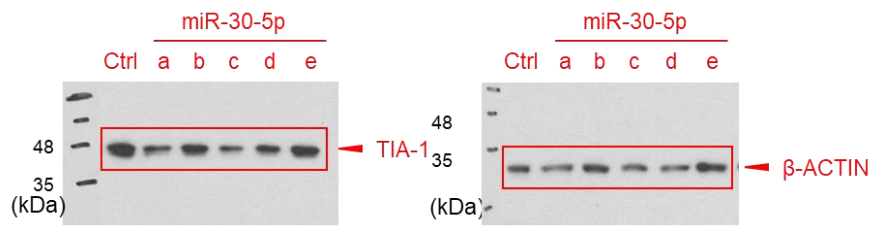

Fig. 5F

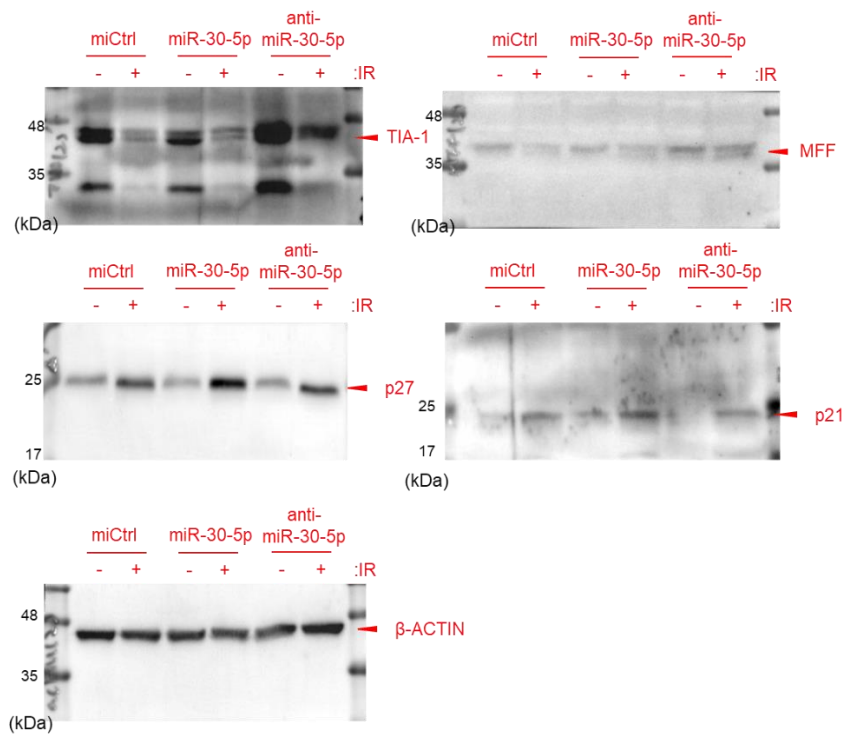

Fig. 5G

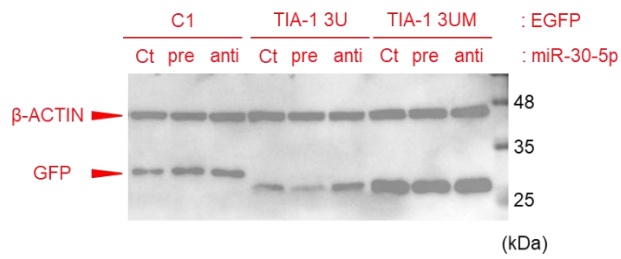

Supplement: Supplementary file 2 — Original Data File [file 41419_2024_6797_MOESM2_ESM.pdf]
